# Construction and Application of Indirect Competitive Enzyme-Linked Immunosorbent Assay for Acetamiprid in Traditional Chinese Medicine

**DOI:** 10.3390/toxics13110982

**Published:** 2025-11-15

**Authors:** Tingting Zhou, Biao Zhang, Xuan Xie, Yuanxi Liu, Hailiang Li, Hongyu Jin, Yongqiang Lin, Feng Wei, Ying Wang

**Affiliations:** 1 Institute for Control of Chinese Traditional Medicine and Ethnic Medicine, National Institutes for Food and Drug Control, Beijing 102629, China; tting_zhou@163.com (T.Z.); xiexuan0525@163.com (X.X.); liuyuanxi@nifdc.org.cn (Y.L.); lihailiang@nifdc.org.cn (H.L.); jhyu@nifdc.org.cn (H.J.); linyongqiang@nifdc.org.cn (Y.L.); weifeng@nifdc.org.cn (F.W.); 2 School of Traditional Chinese Pharmacy, China Pharmaceutical University, Nanjing 211198, China; 3 Key Laboratory of Microbiological Metrology, Measurement & Bio-Product Quality Security, State Administration for Market Regulation, College of Life Sciences, China Jiliang University, Hangzhou 310018, China; zb@cjlu.edu.cn

**Keywords:** acetamiprid, monoclonal antibody, ic-ELISA, traditional Chinese medicine, residue detection

## Abstract

The contamination of traditional Chinese medicines (TCMs) with neonicotinoid pesticides, notably acetamiprid (ACE), poses a significant challenge to product safety. Conventional detection methods are often hampered by operational complexity, prolonged analysis times, and dependence on sophisticated instrumentation, rendering them impractical for rapid on-site screening. To address these limitations, an indirect competitive enzyme-linked immunosorbent assay (ic-ELISA) was developed for the efficient quantification of ACE residue in TCM matrices. A monoclonal antibody-based ic-ELISA was developed through the synthesis of an ACE antigen. Critical assay parameters—including coated antigen concentration, antibody dilution ratio, and blocking buffer composition—were systematically optimized. The validated protocol was subsequently applied to ACE detection in five representative TCMs. The sensitivity (IC_50_), limit of detection (IC_15_), and detection range (IC_20_-IC_80_) of the developed ic-ELISA for ACE were 13.61 ng/mL, 0.50 ng/mL, and 1.00–150.99 ng/mL, respectively. The ic-ELISA demonstrated good stability and specificity, with cross-reactivity for ACE analogs all below 1.5%. Additionally, the ic-ELISA for ACE achieved recoveries of 86.87–104.80% in spiked TCM samples (Lonicerae Japonicae Flos, Lycii Fructus, Bulbus Lilii, Citri Reticulatae Pericarpium, and Jasminum sambae Flos), with relative standard deviations (RSDs) of 3.33–12.05%. The recovery rate of ic-ELISA was verified to be in good consistency with that of high-performance liquid chromatography (86.09–102.10%), indicating that ic-ELISA has acceptable accuracy and precision. This approach is simple and sensitive, making it suitable for the rapid quantitative detection of ACE residues in TCM products. It also provides technical references for the development of ic-ELISA for other small-molecule contaminants.

## 1. Introduction

Acetamiprid (ACE), a first-generation neonicotinoid, is widely used not only in agricultural pest control but also in disease and pest prevention during large-scale cultivation of traditional Chinese medicines (TCMs) owing to its high insecticidal efficiency, low toxicity, and broad pesticidal spectrum [1,2,3,4]. However, growing research has raised concerns regarding its possible hazards to mammalian health. ACE can be absorbed by mammals through the skin, mouth, and respiratory system, and it has been demonstrated in experimental models to be genotoxic, cytotoxic, and reproductively toxic [5]. Therefore, many countries have enacted regulatory measures to reduce the negative effects of ACE on human health. For example, the French government passed legislation in 2016 prohibiting the use of ACE and four other neonicotinoid insecticides [6]. The European Food Safety Authority defined maximum residue limits (MRLs) for ACE in numerous crops such as plums and eggplants at 0.04–0.4 mg/kg [7]. The Chinese Ministry of Agriculture and Rural Affairs also issued the national regulations ‘GB 2763-2021 Standard for Maximum Residue Limits of Pesticides in Food’, which establishes the MRLs for ACE in vegetables, fruits, grains, and other foods at 0.01–15 mg/kg [8].

Traditional medicines can help prevent and treat a variety of diseases. According to a World Health Organization report, almost 80% of the world’s population relies on traditional medicines for primary healthcare, particularly TCMs [9]. Beyond clinical uses, TCMs are widely used in the manufacturing of functional foods [10], food additives [11], and cosmetics [12], resulting in continual growth in the TCM industry’s scale and market demand. As natural TCM resources become increasingly scarce, artificial cultivation has emerged as the primary source of TCMs. Pesticides are frequently used in large-scale cultivation to control pests and illnesses, raising worries about pesticide residual contamination in TCM products.

Previous studies have shown that neonicotinoid insecticides, particularly ACE, are highly polluted in TCMs. Wang et al. [13] used HPLC–MS/MS and GC–MS/MS to screen 168 pesticides in 1017 samples of 10 TCMs. Their findings showed that ACE had a detection rate of 17%. Wang et al. [14] analyzed 109 samples of five TCMs with LC–MS/MS to detect 10 neonicotinoid pesticides and their 10 major hazardous metabolites. Ninety (82.57%) of the samples tested positive for neonicotinoid residues, with concentrations ranging from 0.26 to 139.28 μg/kg. ACE had the highest detection rate (77.06%) and maximum concentration (≤85.95 μg/kg). Gao et al. [15] used UPLC–MS/MS to detect residues of 15 neonicotinoid pesticides and their metabolites in 15 batches of Lycium. Their findings showed an overall neonicotinoid detection rate of 73.3%, with ACE again dominating—exhibiting the highest detection rate (60%) and concentration range (0.0306–1.09 mg/kg). To address ACE overuse and ensure TCM safety, the 2025 edition of the Chinese Pharmacopoeia has established specific MRLs for ACE in Bulbus Lilii, Lycii Fructus, and Lonicerae Japonicae Flos of 0.05 mg/kg, 2 mg/kg, and 15 mg/kg, respectively [16], and the significant difference in these MRLs is mainly related to field experiments. Therefore, the development of reliable, efficient detection methods for ACE in TCMs is urgently needed to enforce regulatory standards and safeguard the safety of TCM products for consumers.

Currently, instrumental analytical techniques are the major method for detecting ACE residues in TCMs, with common approaches including UPLC–MS/MS [17], HPLC [18,19], and LC–HRMS [20]. These approaches have considerable advantages, including wide applicability, high separation efficiency, outstanding sensitivity, and exceptional reproducibility. However, sample preparation procedures are complex and time-consuming, operation requires well-trained professional personnel, and the associated instruments are large and expensive. These problems contribute to high testing costs [21], limiting large-scale general deployment and making them unable to meet the requirements for rapid screening of large batches of TCMs at grassroots-level quality control sites. In recent years, ELISA—based on specific antibody–antigen recognition interactions—has emerged as one of the most promising trace detection techniques for small molecules due to its advantages of simple operation, high sensitivity, low detection cost, and compatibility with on-site real-time testing [22,23,24]. ELISA has matured in the detection of pesticide residues in food and agricultural goods, with researchers developing ELISA for ACE detection in pollen [25], vegetables [26], and fruits [27]. However, TCM samples present unique challenges due to their complex composition and wide range of ACE residue levels. The specificity and detection range of ELISA methods for ACE in TCMs still need to be optimized to fully meet the practical demand for rapid, reliable residue detection.

Despite the growing concerns over ACE contamination in TCMs, the development of high-performance ELISA remains notably understudied. To address this critical gap and meet the urgent need for rapid ACE screening to ensure TCM product safety and regulatory compliance, we developed an ic-ELISA for ACE with both high sensitivity and a broad linear range through the employment of self-prepared monoclonal antibodies and the systematic optimization of key assay parameters. The developed ic-ELISA was successfully employed to rapidly detect ACE residues in Lonicerae Japonicae Flos, Lycii Fructus, Bulbus Lilii, Citri Reticulatae Pericarpium, and Jasminum sambae Flos. This work not only provides a reliable analytical tool for ACE monitoring in TCMs, but also establishes a methodological framework that could be adapted for detecting other neonicotinoid contaminants, thereby contributing to enhanced quality control in herbal medicine production and distribution.

## 2. Materials and Methods

### 2.1. Materials and Apparatus

Ovalbumin (OVA), bovine serum albumin (BSA), urea peroxide, N-hydroxysuccinimide (NHS), EDC (1-(3-dimethylaminopropyl)-3-ethylcarbodiimide hydrochloride), 3,3′,5,5′-tetramethylbenzidine (TMB), dimethyl sulfoxide (DMSO), sodium bicarbonate (NaHCO_3_), skimmed milk powder (SMP), and ethanol were purchased from Sigma Aldrich (Shanghai) Trading Co., LTD. (Shanghai, China). Macklin Biochemical Co., Ltd. (Shanghai, China) provided Tween-20. Jackson Immunoresearch Laboratories (West Grove, PA, USA) provided Goat anti-mouse HRP. Shanghai Aladdin Biochemical Technology Co., Ltd. (Shanghai, China) provided ACE, imidacloprid, alatzin, dinotefuran, nitenpyram, carbendazim, chlorpyrifos, omethoate, trichlorfon, thiacloprid, methanol, and other reagents. TCM materials (are shown in Figure S1): Lonicerae Japonicae Flos (LJF), Lycii Fructus (LF), Bulbus Lilii (BL), Citri Reticulatae Pericarpium (CRP), and Jasminum sambae Flos (JSF) were purchased from the market (Beijing, China).

Corning Incorporated (New York, NY, USA) provided 96-well EIA/RIA plates. Shimadzu (Shanghai, China) provided UV-visible spectrophotometer UV-1800. Waters (Shanghai, China) provided XTerra MS C18 column and Arc HPLC System with 2998 PDA Detector. Thermo Fisher Scientific Co. (Waltham, MA, USA) provided Varioskan LUX multimode microplate reader.

### 2.2. Preparation of ACE Hapten and Antigen

**Figure 1:**
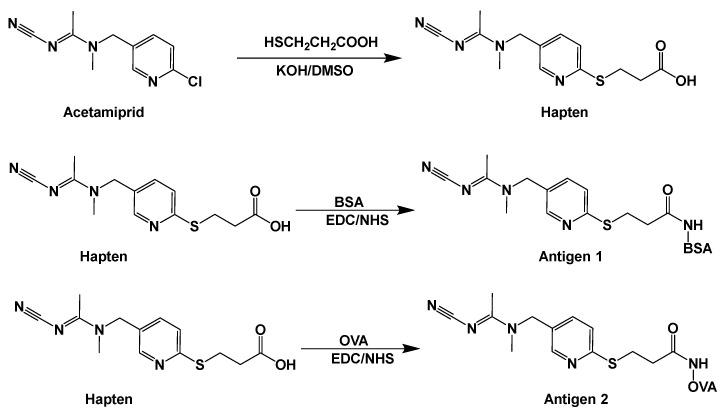
Schematic diagram of the synthesis route for ACE hapten and antigen.

The ACE hapten and antigen synthetic route is shown in Figure 1. Firstly, a mixture containing 2.00 g ACE and 0.9 g KOH was dissolved in 3.0 mL DMSO, followed by the addition of 10 mL DMSO containing 0.84 g β-mercaptopropionic acid which was slowly dropped into the reaction mixture. After stirring at ambient temperature for 12 h, the mixture was filtered and concentrated to yield a yellow viscous liquid, which was then dissolved in 20.0 mL ultrapure water, with the pH of the solution adjusted to 3 using 2.0 mol/L HCl. Then, the aqueous phase was extracted twice with 10.0 mL ethyl acetate each time, and the organic phase was combined and washed twice with ultrapure water. The extracted solution was dried with anhydrous sodium sulfate and concentrated, and the crude product was recrystallized from methanol to obtain ACE hapten as a solid.

One hundred millimoles of the ACE hapten was dissolved in 2.0 mL DMSO. A 1.5 mL DMSO containing 0.1 moL NHS and 0.1 moL EDC was then added to the ACE hapten solution, and the reaction mixture was magnetically stirred at ambient temperature under a sealed condition for 7 h to overnight. After centrifuging at 8000 r/min for 5 min, the supernatant was slowly added into 2.0 mL of 15 mg/mL BSA (or OVA) solution, and the conjugation reaction was carried out by magnetic stirring at room temperature for 12 h. Upon completion of the conjugation reaction, dialysis was performed against 0.01 mol/L PBS solution (pH 7.4) at 4 °C with constant stirring for 4 days, with the dialysate renewed every 12 h. Finally, the ACE antigen was obtained and stored at −20 °C for subsequent use.

### 2.3. Preparation of ACE Monoclonal Antibodies

The animal immunization protocol (Table S1) and hybridoma cell preparation and screening, as well as mouse ascites production and antibody purification procedures, are described in detail in the Supplementary Materials.

### 2.4. Optimization of Working Parameters

Working parameters critically influence ic-ELISA performance. To achieve optimal sensitivity and detection limits, we systematically optimized the following key parameters: the concentration of coated antigen (0.05, 0.10, 0.15 μg/well), a series of dilution coefficients of anti-ACE antibodies (1:8000, 1:12,000, 1:16,000, 1:32,000, 1:64,000, 1:128,000, 1:256,000), and the type of blocking solution (0.4% SMP, 0.8% SMP, 0.4% BSA, 0.8% BSA, 0.4% gelatin, 0.8% gelatin). The HRP-conjugated secondary antibody was used at a fixed dilution of 1:1000. The limit of detection (LOD, calculated as IC_15_) of ACE served as the primary evaluation indicator.

### 2.5. Development of ic-ELISA

The development of the ACE ic-ELISA drew upon the prior work of Sheng et al. [28], with the principal steps outlined as follows: First, each well was coated with the 100 µL ACE coating antigen solution and incubated at 4 °C overnight. After coating, the plate was washed three times with PBST (0.01 mol/L PBS with 10% Tween-20) to remove unbound antigen. Then, each well was blocked with 200 μL of 0.4% SMP solution and incubated at 37 °C for 1 h to block nonspecific binding sites. Once the blocking process was completed, the wells were cleaned again to eliminate residual blocking buffer. For the competitive reaction step, each well was added with 50 µL ACE standard or TCM sample solution and 50 µL ACE antibody solution (the blank well was added with 100 μL of PBS to correct for the TMB spontaneous color reaction in the absence of ACE antibody, HRP), after which the microwell plate was incubated at 37 °C for 1 h to allow the antigen–antibody reaction to occur. Following this reaction, the wells were cleaned again to remove unbound substances. Then, each well was added with 100 μL of the 1000-fold diluted HRP-labeled secondary antibody solution, and the plate was incubated at 37 °C for 30 min to enable the binding of the primary antibodies to the secondary antibodies. After another round of cleaning to eliminate excess secondary antibody, the plate was added with 0.2 mM TMB substrate solution (100 µL/well) and incubated at 37 °C for 15 min. Finally, the reaction in each well was terminated by adding 50 µL of 1.25 mol/L H_2_SO_4_ solution. The absorbance value at 450 nm was determined utilizing multimode microplate reader. S-shaped standard curve was fitted using ACE concentration on the x-axis and inhibition rate on the y-axis. The inhibition rate was calculated through the following formula.

Inhibition rate (%) = (*A* − *A_i_*)/(*A* − *A*_0_) × 100

where *A*_0_, *A_i_*, and *A* correspond to the absorbance values of wells treated as follows: 100 µL PBS solution added for *A*_0_; 50 µL ACE antibody combined with 50 µL different concentrations of ACE solution for *A_i_*; and 50 µL ACE antibody mixed with 50 µL PBS solution for *A*.

### 2.6. Specificity Evaluation of ic-ELISA

Method specificity is one of the key indicators for evaluating ic-ELISA and was defined as the ability to specifically recognize the target compound to bind to the antibody. By adding ACE at a concentration of 100.0 ng/mL, structural or functional analogs of ACE (imidacloprid, alatzin, dinotefuran, nitenpyram, carbendazim, chlopyrifos, omethoate, trichlorfon, thiacloprid) at 1000 ng/mL, as well as positive (containing 100.0 ng/mL ACE) and negative (not containing ACE) mixed solutions, can be used to evaluate method specificity. The cross-reactivity was defined by the ratio of the IC_50_ value of ACE to that of other analogs, and its calculation formula was set as follows:

Cross-reactivity (%) = 100 × IC_50_ (ACE)/IC_50_ (other analogs)

### 2.7. Application Evaluation of ic-ELISA

The stability and accuracy of ic-ELISA were evaluated by detecting ACE in samples of LJF, LF, BL, CRP, and JSF. The sample preparation procedure for ic-ELISA is as follows: 2.0 g sample and 10.0 mL extraction solvent (ethanol: water = 1:9 (*v*/*v*)) were placed in a 50 mL centrifuge tube. The mixture was shaken vigorously for 3 min and then left to stand for 20 min. Subsequently, 5.0 mL of supernatant was transferred to another centrifuge tube as the test solution. For actual sample analysis, the above-mentioned test solution needs to be appropriately diluted before use. The sample preparation procedure and key parameters of HPLC are provided in Supplementary Materials.

## 3. Results and Discussion

### 3.1. Characterization of Antigens and Screening of Antibodies

**Figure 2:**
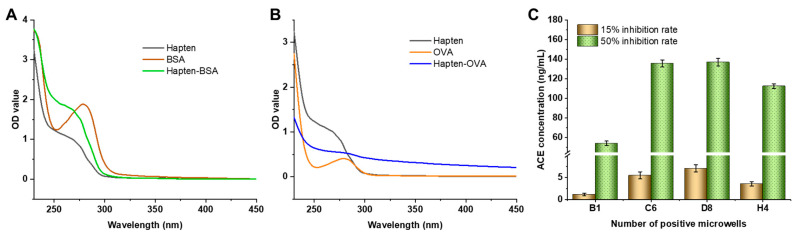
Ultraviolet absorption spectra of ACE–BSA (**A**) and ACE–OVA (**B**). Screening of positive hybridoma cell lines (**C**). Error bars represent the standard deviation (SD, *n* = 3).

The conjugation efficiency of ACE hapten to ovalbumin (ACE–OVA) and bovine serum albumin (ACE–BSA) was quantitatively assessed by UV–Vis spectroscopy. As illustrated in Figure 2A,B, the characteristic absorption peaks of the conjugates exhibited distinct bathochromic shifts compared to BSA and OVA, with the resultant spectra demonstrating intermediate wavelengths between the hapten and protein absorption maxima. This observed hypsochromic shift, coupled with alterations in peak morphology, is consistent with established conjugation characteristics reported in the literature [29], thereby confirming successful covalent linkage between the hapten and carrier proteins. Subsequent immunization studies provided additional validation of the successful conjugation of the hapten to the carrier proteins.

Following three iterative cycles of subcloning, four stable monoclonal antibody-producing hybridoma cell lines (designated B1, C6, D8, and H4) were obtained. As shown in Figure 2C, under conditions of 0.1 μg/well coated antigen and 1:32,000 antibody dilution coefficient, the IC_15_ for the four antibody groups were 1.2 ng/mL, 5.5 ng/mL, 7.1 ng/mL, and 3.6 ng/mL, with corresponding IC_50_ of 54.3 ng/mL, 135.6 ng/mL, 136.9 ng/mL, and 112.6 ng/mL, respectively. Among the four cell lines, the B1 cell line (immunized with ACE–BSA) produced antibodies with the lowest IC_15_ and IC_50_, and was selected for subsequent development of ic-ELISA.

### 3.2. Optimization of ic-ELISA Conditions

**Figure 3:**
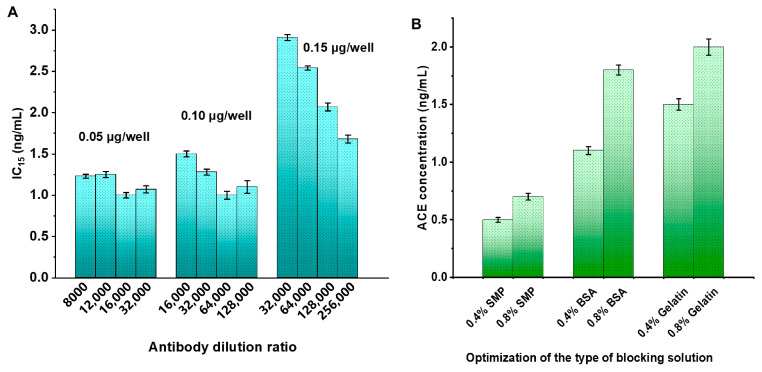
Optimization of coated antigen concentration and antibody dilution factor (**A**) and blocking solution (**B**). Error bars represent the standard deviation (SD, *n* = 3).

The concentration of the coated antigen, the dilution factor of the anti-ACE antibody, and the type of blocking solution are critical factors influencing the performance of ic-ELISA [30]. Systematic optimization of these variables was conducted to enhance the assay sensitivity of ic-ELISA. As presented in Figure 3A, reducing the ACE coated antigen concentration from 0.15 μg/well to 0.05 μg/well generally decreased the IC_15_ value. When the ACE coated antigen was at a concentration of 0.05 μg/well and the ACE antibody had a dilution factor of 1:16,000, the constructed ic-ELISA exhibited the lowest IC_15_ value of 1.0 ng/mL. Further optimization was performed to evaluate the impact of blocking solutions on assay performance (Figure 3B). Comparative analysis revealed that SMP exhibited the highest blocking efficiency, yielding an IC_15_ of 0.5 ng/mL at 0.4% (*w*/*v*), compared to 1.1 ng/mL for BSA and 1.5 ng/mL for gelatin at the same concentration. Notably, 0.4% blocking solutions consistently outperformed their 0.8% counterparts, suggesting that excessive blocking agent concentration may hinder antibody–antigen interactions. Thus, 0.4% SMP was selected as the optimal blocking solution for subsequent ic-ELISA.

### 3.3. Establishment of ic-ELISA Standard Curve

**Figure 4:**
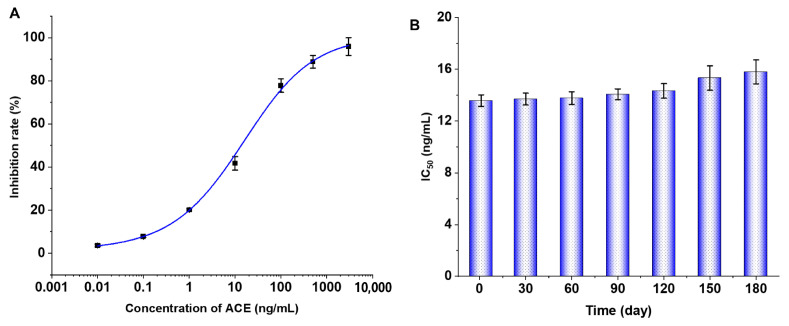
ACE sigmoidal standard curve (**A**) and stability evaluation (**B**) of the ic-ELISA. Error bars represent the standard deviation (SD, *n* = 3).

Under optimal working conditions, the S-shaped standard curve of ic-ELISA for ACE was established with the following equation: y = 1.3549 + (100.7262 − 1.3549)/[1 + (21.6648/x)^0.5922^]^0.8501^ (R^2^ = 0.9969). Calculations showed that ic-ELISA had a sensitivity (IC_50_) of 13.61 ng/mL, LOD (IC_15_) of 0.50 ng/mL, and a detection range (IC_20_-IC_80_) of 1.00–150.99 ng/mL (Figure 4A). To evaluate the stability of the established ic-ELISA, 96-well plates were blocked and stored at −20 °C, and the performance of ic-ELISA was monitored over 180 days. As shown in Figure 4B, the IC_50_ increased slightly from 13.6 ng/mL to 15.8 ng/mL, and statistical analysis revealed no significant difference in sensitivity (*p* > 0.05), confirming that the ic-ELISA exhibits excellent stability, maintaining reliable performance over 180 days.

### 3.4. Evaluation of Specificity

**Figure 5:**
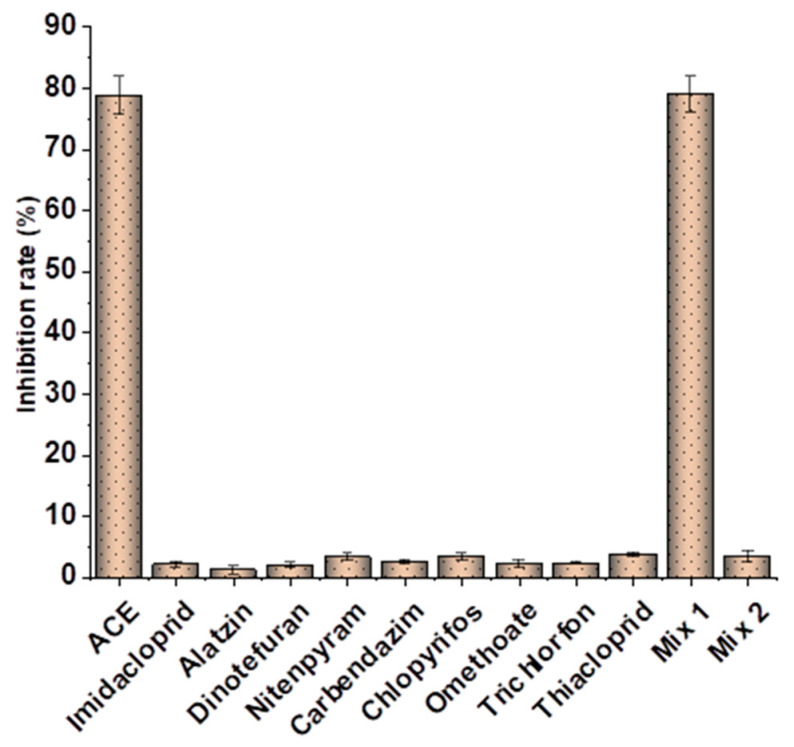
Specificity evaluation of ic-ELISA. Error bars represent the standard deviation (SD, *n* = 3).

To further verify the specificity of the established ic-ELISA, single standard substances (100.0 ng/mL ACE, 1000.0 ng/mL each of structurally/functionally analogous compounds) and mixed standard solutions including Mix 1 (containing 100.0 ng/mL ACE) and Mix 2 (containing no ACE) were tested. Results are shown in Figure 5. The inhibition rates of 100.0 ng/mL ACE and Mix 1 (containing 100.0 ng/mL ACE) were 78.8% and 79.0%, respectively. In contrast, inhibition rates of other standard solutions were all less than 4.0%, with cross-reactivity consistently less than 1.5% (Table 1). This confirms that the prepared ACE antibody possesses high specificity, being capable of recognizing only ACE. Compared with previously reported ACE antibodies [31], the antibody in this study exhibits lower cross-reactivity with thiamethoxam and thiacloprid.

### 3.5. Application in Actual Samples

The absence of ACE residues in the test samples was confirmed through parallel analysis using both HPLC and ic-ELISA, qualifying these samples as appropriate blank controls for subsequent experiments. For the validation of the developed ic-ELISA’s reliability, ACE was spiked into samples at three distinct concentrations (5, 50, and 500 µg/kg). For actual sample analysis, the sample extract was diluted and tested by this method. As shown in Table 2, the recoveries of ACE by the ic-ELISA ranged from 86.87% to 104.80% with RSDs of 3.33–12.05%. These results showed excellent agreement with recovery values (86.09–102.10%) with RSDs (1.55–7.40%) using HPLC. The outcomes obtained from ic-ELISA showed consistency with HPLC results, with a correlation coefficient (R^2^) of 0.9980 (Figure S2). These results confirm that the established ic-ELISA possesses favorable accuracy, and it is applicable for detecting ACE in various parts of medicinal plants, including LJF (flower), JSF (flower), LF (fruit), BL (bulb), and CRP (fruit peel).

### 3.6. Comparison with Other Immunoassays

Many similar immunoassay reports were collected for the purpose of comparative analysis. The comparison results (sensitivity, detection range, cross-reactivity, applicable matrix, and pre-treatment) are shown in Table 3. First, this study is the first to develop an ic-ELISA method for detecting ACE in TCMs, while previous studies have primarily focused on food and agricultural products, such as Chinese chives [32] and peaches [33]. Second, the established conventional HRP-based ic-ELISA exhibits a sensitivity and detection range essentially consistent with those studies that use novel signal-amplifying materials such as peroxidase-like bimetallic nanoenzyme [34] or chemiluminescence enzyme [35]. Future work will explore the application of novel labeling materials such as nanoenzymes and artificial enzymes in TCM detection. Third, during sample preparation, ethanol was used for pesticide extraction instead of methanol or acetonitrile as in other ACE immunoassays [36,37], featured by lower toxicity, reduced solvent consumption, and simpler pre-treatment steps.

## 4. Conclusions

Based on the monoclonal antibody that had been prepared, the optimized ic-ELISA for the neonicotinoid insecticide ACE was successfully constructed and demonstrated a sensitivity (IC_50_) of 13.61 ng/mL, LOD (IC_15_) of 0.50 ng/mL, and detection range (IC_20_–IC_80_) of 1.00–150.99 ng/mL. The constructed ic-ELISA detected the other pesticides, showing cross-reactivity less than 1.5%. A comparison between the results of the proposed ic-ELISA and HPLC revealed strong consistency, with their correlation coefficient (R^2^) reaching 0.9980. The developed ic-ELISA was successfully applied to detecting ACE in five TCMs: LJF, LF, BL, CRP, and JSF. In conclusion, this rapid, accurate, and cost-effective ic-ELISA provides a practical solution for monitoring ACE residues in TCMs, supporting quality control and safety assurance. Furthermore, the methodological framework established here offers a valuable reference for developing ic-ELISAs targeting other small-molecule contaminants, including veterinary drug residues, biotoxins, and illegal additives in herbal products.

## Figures and Tables

**Table 1:** Cross-reactivity of ic-ELISA.

Name	Structural Formula	IC_50_ (ng/mL)	Cross-Reactivity (%)
ACE	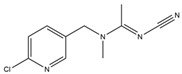	13.61	100.0
Imidacloprid	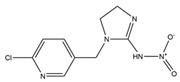	>1000	<1.5
Alatzin	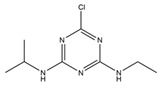	>1000	<1.5
Dinotefuran	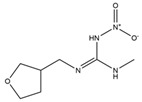	>1000	<1.5
Nitenpyram	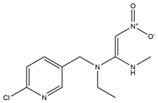	>1000	<1.5
Carbendazim	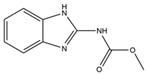	>1000	<1.5
Chlopyrifos	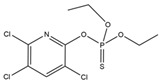	>1000	<1.5
Omethoate	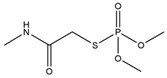	>1000	<1.5
Trichlorfon	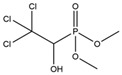	>1000	<1.5
Thiacloprid	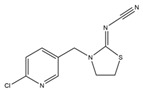	>1000	<1.5

**Table 2:** ACE determination results through ic-ELISA and HPLC in real samples (*n* = 3).

TCM Sample	Spiked Level (μg/kg)	ic-ELISA			HPLC		
		Mean (μg/kg)	Recovery (%)	RSD ^a^ (%)	Mean (μg/kg)	Recovery (%)	RSD (%)
LJF	5.0	4.69	93.87	7.46	4.45	88.93	2.02
	50.0	50.56	101.13	8.25	51.05	102.10	1.80
	500.0	478.53	95.71	8.28	486.23	97.25	5.83
JSF	5.0	4.90	98.00	3.33	5.05	100.93	3.95
	50.0	49.27	98.53	5.89	49.93	99.87	2.02
	500.0	478.07	95.61	9.13	509.61	101.92	3.23
LF	5.0	4.34	86.87	8.93	4.23	84.53	6.28
	50.0	43.84	87.69	12.05	43.73	87.47	7.40
	500.0	444.03	88.81	9.64	430.43	86.09	6.42
CRP	5.0	4.67	93.33	8.81	4.58	91.53	6.06
	50.0	52.40	104.80	7.56	50.50	101.00	5.44
	500.0	507.50	101.5	7.01	481.80	96.36	5.81
BL	5.0	4.81	96.20	6.71	4.49	89.80	1.55
	50.0	49.80	99.60	7.56	50.27	100.53	5.60
	500.0	465.87	93.17	9.82	505.47	97.26	2.71

^a^ RSD, relative standard deviation.

**Table 3:** Comparison of the ACE immunoassay.

Method	Sensitivity	Detection Range	Cross-Reactivity	Matrix	Sample Preparation	Reference
AuNPs-LFIA	Cut-off value 10.0 ng/mL	— ^a^	Thiacloprid(30%), Imidaclothiz(30%), Imidacloprid(3%)	Tea	Extracted with 100% methanol and diluted with PBST	[31]
dc-ELISA	IC_50_ 0.16 ng/mL	0.043–0.6 ng/mL	Thiacloprid(43.8%), IM-2-1(2.1%)	Chinese chive	Extracted with water and diluted with water	[32]
ELISA	I_50_ 0.6 ng/g	0.18–3 ng/g	Thiacloprid(40%), Imidacloprid(0.62%)	Peach, apple, strawberry, cucumber, eggplant, and tomato	Extracted with 100% methanol and diluted with water	[33]
Au@Pt-assisted ic-ELISA	IC_50_ 25.58 μg/L	1.85–327.19 μg/L	— ^a^	Chinese cabbage, cucumber, and zucchini	Extracted with 100% methanol and diluted with PBS	[34]
ic-CLEIA	IC_50_ 10.24 ng/mL	0.70–96.31 ng/mL	Clothianidin(8.63%), Thiacloprid(4.79%), Nitenpyram(1.96%)	Chinese cabbage and cucumber	Extracted with 99.5% acetone and diluted with sub-boiling water	[35]
IFE-IA	SC_50_ 0.04 μg/L	0.002–0.58 μg/L	Thiacloprid(36.4%)	Soil, pear, wheat, and cucumber	Extracted with borate saline buffer (0.14 mol/L) containing 20% methanol and diluted with buffer	[36]
TRFIS	LOD 0.056–0.074 mg/kg	0.25–1.75 mg/kg	— ^a^	Celery cabbage, cauliflower, and baby cabbage	Extracted with 100% methanol and diluted with PBST	[37]
ic-ELISA ^b^	IC_50_ 13.61 ng/mL	1.00–150.99 ng/mL	Thiacloprid(<1.5%), Imidacloprid(<1.5%)	LJF, LF, BL, CRP, and JSF	Extracted with 10% ethanol	This study

^a^ not mentioned in the literature. ^b^ this study.

## Data Availability

The datasets generated for this study are available on request to the corresponding author.

## Supplementary Materials

The following supporting information can be downloaded at https://www.mdpi.com/article/10.3390/toxics13110982/s1, Experimental methods, Figure S1: The TCM materials used in this study; Figure S2: Correlation analysis between ic-ELISA and HPLC detection results; Table S1: Immunization program.
