# Supplementary material for: Construction and Application of Indirect Competitive Enzyme-Linked Immunosorbent Assay for Acetamiprid in Traditional Chinese Medicine
Source: Toxics. 2025 Nov 15;13(11):982. doi: 10.3390/toxics13110982 (PMC12655919; doi:10.3390/toxics13110982)
Supplement: Supplementary file 1 [file toxics-13-00982-s001.zip › toxics-3962956-supplementary.pdf]

Construction and Application of Indirect Competitive Enzyme-linked Immunosorbent Assay for Acetamiprid in Traditional Chinese Medicine

Tingting Zhou[1,2,†], Biao Zhang[3,†], Xuan Xie[1], Yuanxi Liu[1], Hailiang Li[1], Hongyu Jin[1], Yongqiang Lin[1], Feng Wei[1], Ying Wang[1,*]

[1] Institute for Control of Chinese Traditional Medicine and Ethnic Medicine, National Institutes for Food and Drug Control, Beijing 102629, China; tting_zhou@163.com (T.Z.); xiexuan0525@163.com (X.X.); liuyuanxi@nifdc.org.cn (Y.L.); lihailiang@nifdc.org.cn (H.L.); jhyu@nifdc.org.cn (H.J.); linyongqiang@nifdc.org.cn (Y.L.); weifeng@nifdc.org.cn (F.W.)

[2] School of Traditional Chinese Pharmacy, China Pharmaceutical University, Nanjing 211198, China

[3] Key Laboratory of Microbiological Metrology, Measurement & Bio-product Quality Security, State Administration for Market Regulation, College of Life Sciences, China Jiliang University, Hangzhou 310018, China; zb@cjlu.edu.cn (B.Z.)

[*] Correspondence: wangying17@nifdc.org.cn (Y.W.)

[†] These authors contributed equally to this work.

Preparation of Monoclonal Antibodies

1. Animal Immunisation

Twelve 6-week-old female BALB/c mice were randomly divided into two groups for immunisation, with each group targeted to one of the two immunogens (ACE–BSA or ACE–OVA). Each immunogen was diluted in physiological saline, then thoroughly emulsified with Freund's complete adjuvant (FCA) at a 1:1 (v/v). Mice in each group received an intraperitoneal injection of 0.5 mL of the emulsified mixture. The second immunisation was conducted three weeks after the primary one, followed by additional boosters every 14 days thereafter. For boosters, immunogens were emulsified with Freund's incomplete adjuvant (FIA) at 1:1 (v/v), and administered via intraperitoneal injection (0.5 mL per mouse) using the same protocol. Tail vein blood was collected from mice on day 7 after the third, fourth, and fifth immunisations. Collected blood was dissolved in PBS buffer, stored at 4°C, and subjected to ic-ELISA analysis on the same day to determine antibody titres.

The ELISA procedure for serum titre detection was conducted as follows: A 96-well microplate was coated with 0.1 μg/well of ACE antigen and incubated overnight at 4°C; the plate was washed three times with PBST, then 200 μL/well of 0.5% SMP blocking solution was added, and the plate was incubated at 37°C for 50 min, followed by another three washes with PBST. A measurement of 50 μL of ACE standard or PBS buffer (blank control) was added to each well, and 50 μL of serially diluted serum was subsequently added; the plate was incubated at 37°C for 50 min and then washed four times with PBST. 100 μL/well of HRP-labelled secondary antibody was added, and the plate was incubated at 37°C for 30 min. The solution in the wells was discarded; the plate was washed five times with PBST and blotted dry. Substrate mixture AB was added, and

the plate was incubated for 15 min in a 37°C incubator for colour development. The reaction was terminated by adding 50 μL/well of sulphate stop solution, and the absorbance at 450 nm was measured using a microplate reader. After the fifth immunisation, the BALB/c mouse with the highest serum antibody titre (meeting cell fusion requirements) was selected; a final booster immunisation was performed using the corresponding immunogen (directly diluted in physiological saline), and detailed experimental parameters are shown in Table S1.

2. Preparation and Screening of Positive Hybridoma Cells

2.1 Cultivation of Myeloma Cells and Acquisition of Spleen Cells

Cryopreserved SP2/0 myeloma cells were thawed in a 37°C water bath, then centrifuged at 1000 rpm for 5 min to remove the supernatant. The cell pellet was resuspended in complete culture medium and transferred to culture flasks for expansion. After 6–8 h of incubation in a cell culture incubator, the medium was replaced to eliminate dead cells and residual DMSO, reducing the toxic effects of organic reagents on the cells. Cell status was monitored regularly; once cells showed uniform size, compactness, and rounded morphology, they were transferred to new culture flasks for further expansion. When cells uniformly covered over 90% of the flask surface, they were detached using DMEM incomplete medium and prepared for cell fusion.

2.2 Extraction of Splenocytes and Feeder Cells

Mice with the optimal immune response were selected for final booster immunisation, then euthanised via cervical dislocation and immersed in 75% ethanol for 5–10 min for disinfection. In a laminar flow cabinet, the mice were fixed, and the skin and peritoneum on the left flank were incised with sterile medical scissors. Epithelial tissue was dissected to isolate the spleen, which

was rinsed in DMEM incomplete medium to remove adherent fat and connective tissue. The spleen was placed in a 100-mesh nylon sieve containing 10.0 mL DMEM incomplete medium and ground to prepare a splenocyte suspension. The suspension was transferred to a 50 mL centrifuge tube, centrifuged at 1000 rpm for 5 min, and the pellet was washed once with complete medium followed by a second centrifugation.

After alcohol disinfection, BALB/c mice were dissected on a sterile platform. The skin layer was lifted to expose the peritoneum, which was disinfected with an alcohol swab. Then, 8.0 mL of DMEM incomplete medium was injected into the abdominal cavity; after repeated aspiration, the peritoneal fluid was collected and centrifuged at 1000 rpm for 5 min. The supernatant was discarded, the cell pellet was resuspended in HAT medium, and 100 μL of the cell suspension was seeded into each well of a 96-well microplate.

2.3 Cell Fusion Culture and Primary Screening

Splenocytes and exponentially growing SP2/0 myeloma cells were mixed at a 10:1 ratio in a centrifuge tube, supplemented with DMEM medium to 25.0 mL, and thoroughly mixed. The mixture was centrifuged at 1000 rpm for 5 min, and the supernatant was discarded. In a 37°C water bath, 1.0 mL of 45% PEG 2000 was added to the tube within 1 min with gentle shaking (initially rapid, then slow), followed by a 1 min standing period. Over the next 3 min, 2.0 mL of pre-warmed complete medium was added (initially rapid, then slow); over the subsequent 5 min, 25.0 mL of pre-warmed DMEM incomplete medium was added to terminate PEG-induced fusion. After 10 min of standing, the mixture was centrifuged at 800 rpm for 5 min; the supernatant was removed, and the cell pellet was resuspended in 40.0 mL of HAT medium. A 150.0 μL aliquot of

this suspension was added to each well of the microplates pre-seeded with feeder cells, followed by incubation in a 37°C, 5% CO_2 incubator.

Post-fusion, the cultures were inspected for contamination via inverted microscope after overnight incubation. On day 3 post-fusion, half the medium in each well was replaced with HAT medium (first medium change). On day 7 post-fusion, full replacement with HAT medium was performed (second medium change). After another 2–3 days, partial replacement with HT medium was conducted (third medium change). Two weeks post-fusion, full replacement with HT medium was carried out (fourth medium change), and the medium was switched to complete culture medium thereafter. Hybridoma colonies (large, round, transparent cells) appeared on days 15–20. Once colonies occupied one-third of the well, antibody titre assays were performed. Cell lines with high supernatant titres and high inhibition rates were selected for subsequent experiments

2.4 Cloning of Positive Hybridoma Cells

Positive hybridoma cell lines were selected from the 96-well plate, transferred via pipette to a 24-well plate containing complete culture medium, and cultured continuously. After 8 h of incubation, cell adhesion was observed. The finite dilution method was used to dilute the cell populations with complete medium. A measurement of 250 μL of the diluted cell suspension was added to each well of a new plate, ensuring one cell per well. Cultivation was conducted in a 37°C, 5% CO_2 incubator for 5 days, and cell cloning was monitored using a microscope. Once cell colonies occupied one-third of the well, antibody titre testing was performed. Three to five cell lines with superior performance were selected for expansion and subjected to one–two rounds of subcloning to confirm their monoclonal origin. Stable positive cell lines were identified via ELISA

analysis of cell supernatants. Finally, these cell lines were transferred to culture flasks for further expansion, followed by cryopreservation and ascites production.

2.5 Cryopreservation and Recovery of Hybridoma Cells

Logarithmic phase hybridoma cells with robust growth in culture flasks were processed for cryopreservation: the supernatant was removed and the cells were washed with medium and resuspended in complete medium via pipetting. The cell suspension was transferred to centrifuge tubes and centrifuged at 1000 rpm for 5 min and the supernatant was discarded. Each cell pellet was resuspended in 2.0 mL of complete medium supplemented with fresh bovine serum, then aliquoted into two cryovials; 0.2 mL DMSO was added to each vial. After pre-cooling at 4°C for 30 min and pre-freezing at -20°C for 120 min, the vials were stored overnight in an ultra-low temperature freezer before final transfer to liquid nitrogen with proper labelling.

Labelled hybridoma cell vials were retrieved from liquid nitrogen, thawed in a 37°C water bath with gentle manual agitation, and removed when slight ice crystals remained. The suspension was centrifuged at 1000 rpm for 5 min, the supernatant discarded, and the pellet resuspended in complete medium. The cells were transferred to a 24-well plate for 8 h of culture, followed by medium replacement to remove dead cells and residual organic reagents. Cell morphology was monitored using an inverted microscope; once cells showed uniform size, rounded translucent appearance, and compact confluence, they were transferred to culture flasks for further cultivation.

3. Preparation of Mouse Ascites and Antibody Purification

To enhance ascites production, each mouse was injected with 500 μL of FIA. Three days later, logarithmic phase hybridoma cells were centrifuged and resuspended in 2 mL DMEM and 500 μL

of the cell suspension was injected per mouse. After two weeks, ascites was collected via abdominal puncture and centrifuged at 4000 rpm for 10 min and the supernatant was stored at 4°C for immediate purification.

Monoclonal antibodies were purified using the octanoic acid–ammonium sulphate precipitation method: Ascites supernatant was centrifuged at 12000 rpm for 5 min; three volumes of 0.06 mol/L sodium acetate solution (pH 4.0) were added, mixed thoroughly, and the pH was adjusted to 4.5 with 0.1 mol/L HCL. Octanoic acid (35 μL per mL of ascites) was added, stirred for 30 min, and the mixture was refrigerated at 4°C for 60 min. After centrifugation at 10000 rpm for 30 min, the supernatant was collected, filtered through filter paper, and supplemented with 10% volume of 0.1 mol/L PBS (pH 7.4), with pH adjusted to 7.4 using NaOH. Following 15 min of pre-cooling, 0.28 g/mL ammonium sulphate was slowly added with stirring and the mixture was reacted for 15 min and stood at 4°C for 2 h. After centrifugation at 10000 rpm for 30 min, the precipitate was resuspended in 0.01 mol/L PBS (pH 7.4), dialyzed against the same buffer for 48 h, aliquoted, and stored at -20°C.

HPLC Validation

The samples were pretreated for HPLC as follows. The 2.0 g powder of Chinese herbal medicine samples were transferred into 50 mL centrifuge tubes with 15.0 mL 1% acetic acid solution, respectively. The powder solution was completely mixed and allowed to stand for 25 min. Then, 15.0 mL 10% ethyl acetate solution was added into the above solution, and the mixture was shaken for 25 min. 6 g anhydrous magnesium sulphate and 1.5 g anhydrous sodium acetate were added into the mixture. After being vortexed for 5 min, the sample supernatant was collected by centrifugation (6000 rpm, 5 min). A 6 mL sample of supernatant was added into the C_{18} extraction

column for purification, and the collected sample liquid was heated up to nearly dry. Finally, 1.0 mL acetonitrile was added, and the sample solution was treated with a 0.22 μm filter millilitre and stored at 4℃ for later use.

HPLC analysis conditions are listed as follows. The Waters C_{18} reversed phase column (column length 250 mm, inner diameter 4.6 mm, particle size 5 μm) was employed to detect ACE. Mobile phase A: 1% phosphoric acid; mobile phase B: acetonitrile=60%:40%. The flow rate of the mobile phase was 0.8 mL/min. The column temperature was set to 30.0°C and the injection volume was 20.0 μL. The wavelength was set to 254 nm. To simultaneously determine using the ic-ELISA and HPLC methods, three replicates for each spiked concentration were used.

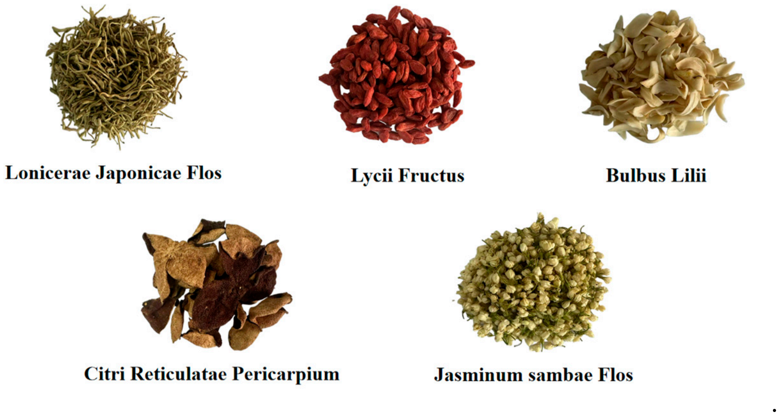

Figure S1. The TCM materials used in this study.

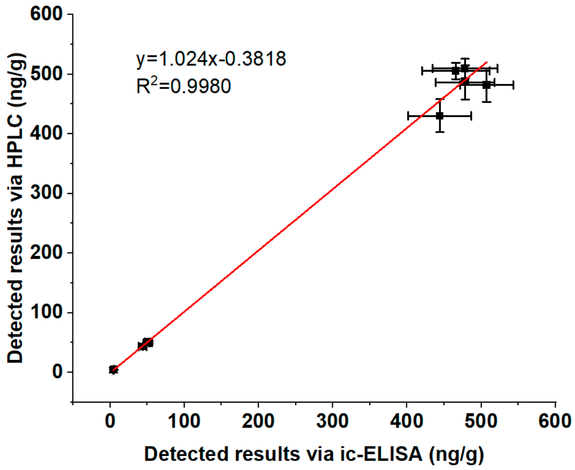

Figure S2 Correlation analysis between ic-ELISA and HPLC detection results.

Table S1. Immunization program.

**Immunisation period**	**Immunogen**	**Immunisation dosage（mg）**
First immunisation	ACE–BSA/ACE–OVA+FCA	0.06
Second immunisation	ACE–BSA/ACE–OVA+FIA	0.1
Third immunisation	ACE–BSA/ACE–OVA+FIA	0.1
Forth immunisation	ACE–BSA/ACE–OVA+FIA	0.1
Fifth immunisation	ACE–BSA/ACE–OVA+FIA	0.1
Booster immunisation	ACE–BSA/ACE–OVA	0.1
